# An open-source high-precision hive for long-term honeybee observation and research

**DOI:** 10.1242/bio.062523

**Published:** 2026-06-30

**Authors:** Jiří Ulrich, Martin Stefanec, Tomáš Rouček, Laurenz Alexander Fedotoff, Harald Pascher, Zdeněk Rozsypálek, Thomas Schmickl, Tomáš Krajník

**Affiliations:** ^1^ Artificial Intelligence Center, Department of Computer Science, Faculty of Electrical Engineering, Czech Technical University in Prague, Prague, 121 35 Praha 2, Czech Republic; ^2^ Artificial Life Lab, Department of Zoology, Institute of Biology, University of Graz, 8010 Graz, Austria

**Keywords:** *Apis mellifera*, Automated observation, Computer vision, Open research

## Abstract

Honeybees have been of interest due to their important role in ecology and agriculture. *Apis mellifera* is a uniquely manageable eusocial insect, defined by its self-organising behaviour, extensive history of domestication and indispensable agricultural role. These attributes, alongside recent high annual losses, necessitate continued scientific research. This research is further accelerated by artificial intelligence and computer vision methods that automatically analyse events in observation hives. However, many findings are not reproducible, even though the methods and models are open source. One of the key problems is the lack of standardisation in the observation-hive setup, leading to significantly different image properties. We present an open-hardware setup design that can be adapted to various research needs and further support the repeatability and transferability of experiments. Using an open-source tracking system, we demonstrate the proposed observation hive on the application of focused observation of an individual honeybee. The hardware designs and software codes are publicly available to the community.

## Teaser

**Summary:** An open-hardware hive system standardises bee observation, enabling consistent data processing across studies. Compatible with standard beekeeping comb frames, it addresses key reproducibility challenges in honeybee research.

## INTRODUCTION

The Western honeybee (*Apis mellifera*) has been subject to human curiosity for hundreds, if not thousands, of years (Kitchell and Resnick, 2000), not only due to its ecological and economic importance (Hung et al., 2018) but also due to the particular eusocial behaviours (Queller and Strassmann, 2003) exhibited by this species. Honeybee colonies can comprise several thousand individuals, which work collectively to maintain the homeostasis of their nest as a whole (Schmickl and Crailsheim, 2004), gather and organise nutrients from the environment (Seeley, 1989) to ensure the survival and growth of the colony and even reproduce as a superorganism through swarming (Allen, 1956). Additionally, honeybees exhibit complex group behaviours, such as temporal polyethism (Seeley, 1982; Seeley and Kolmes, 1991), collective brood care (Schmickl et al., 2003; Sagili and Pankiw, 2009; Siefert et al., 2020), and collective overwintering (Stabentheiner et al., 2010; Barmak et al., 2023), and have even developed a symbolic dance language to communicate information from the environment directly (von Frisch, 1954). These behaviours have all been studied in observational hives.

For nearly three centuries (de Reaumur, 1737), beehives equipped with at least one glass pane have been designed to allow the colony's internal dynamics to be continuously observed. The hives used in such studies vary in size and shape, depending on the study's specific aims, and are often constructed to facilitate the implementation of particular experimental procedures.

Table 1 highlights key ethological studies on honeybees that relied on observation hives. The table is not exhaustive but represents a selection of influential publications that illustrate the breadth of research into honeybee behaviour and social organism studies.

**Table 1.** Overview of key studies using observation hives for honeybee research

Category	Until 1994	1994–2004	2004–2014	2014–2025
Classical observational studies on honeybee behaviour	de Reaumur, 1737; Huber, 1841; von Frisch, 1949, 1954; Allen, 1956, 1960; Wang and Moeller, 1970; Seeley, 1979, 1982; Visscher and Seeley, 1982; Schneider et al., 1986; Seeley and Kolmes, 1991; Seeley, 1992; von Frisch, 1993	Crailsheim et al., 1996; Riessberger and Crailsheim, 1997; Seeley et al., 1998; Rohrseitz and Tautz, 1999; Pernal and Currie, 2001; Gilley, 2001; Schmolz et al., 2002; Michelsen, 2003; Steffan-Dewenter and Kuhn, 2003; Schmickl et al., 2003	Beekman et al., 2004; Gilley and Tarpy, 2005; Biesmeijer and Seeley, 2005; Riley et al., 2005; Mattila and Otis, 2006; Mattila et al., 2008; Johnson, 2008; Kovac et al., 2009; Johnson et al., 2010; Riddell Pearce et al., 2013	Walton and Toth, 2016; Danner et al., 2016; Łopuch and Tofilski, 2017; Ramsey et al., 2018; Hrncir et al., 2019; Klein and Busby, 2020; Siefert et al., 2021; Smith and Peck, 2023
Classical experimental studies on honeybee behaviour	Wenner, 1962; Visscher, 1983; Michelsen et al., 1986a,b; Seeley, 1989; Free et al., 1992; Schneider and McNally, 1992; Kirchner, 1993; Camazine, 1993; Spivak and Gilliam, 1993; Seeley, 1994	Tautz, 1996; Kühnholz and Seeley, 1997; Seeley, 1997; Tautz and Rohrseitz, 1998; Moore et al., 1998; Crailsheim et al., 1999; Weidenmüller and Seeley, 1999; Sullivan et al., 2000; Schmickl and Crailsheim, 2001, 2002; Schneider and DeGrandi-Hoffman, 2002	Schmickl and Crailsheim, 2004; Farina et al., 2005; Grüter et al., 2008; Sagili and Pankiw, 2009; Ohashi et al., 2009; Klein et al., 2010; Rueppell et al., 2010; Bromenshenk et al., 2010; Stabentheiner et al., 2010; Grüter and Ratnieks, 2011; Mattila et al., 2012; Baracchi et al., 2012	Michelsen, 2014; Bienefeld et al., 2015; Ostwald et al., 2016; Siefert et al., 2020; Stefanec et al., 2022; Barmak et al., 2023; Shakeel and Brockmann, 2024; Tokach et al., 2024; Reams et al., 2024
Automated observational or experimental studies on honeybee behaviour			Kimura et al., 2008; Kimura et al., 2011	Wario et al., 2015; Wario et al., 2017; Boenisch et al., 2018; Takahashi et al., 2019; Bozek et al., 2021; Stefanec et al., 2021; Smith et al., 2016, 2022; Žampachů et al., 2022; Gernat et al., 2023; Kongsilp et al., 2024; Ulrich et al., 2024; Blaha et al., 2025

To contextualise the methodological evolution within this field, we categorised these influential works into three distinct observational paradigms. First, classical observational studies (Table 1, top row) relied entirely on human observation without direct intervention, yielding foundational discoveries such as the decoding of the symbolic dance language (von Frisch, 1954). Second, classical experimental studies (Table 1, middle row) involved active manipulation of the colony or hive environment, monitored via human observation.

The third category (Table 1, bottom row) represents a fundamental methodological shift that began in 2008: the transition towards automated and semi-automated observation systems. These studies utilise computer vision and electronic sensors to automatically evaluate experiments or infer complex behavioural metrics, significantly expanding the volume of analysable data. For example, Wario et al. (2015, 2017) utilised webcams and high-resolution cameras with infrared (IR) lamps to automate the detection and decoding of the waggle dance. Boenisch et al. (2018) achieved tracking of all individuals within a colony using 12 MP cameras by affixing physical markers to each animal. Conversely, Bozek et al. (2021) accomplished markerless tracking using 5120×5120-resolution cameras and multi-angle IR LED panels, though this required immense computational overhead. More recently, Blaha et al. (2025) utilised 4K cameras and direct IR lighting to achieve tracking of the queen throughout an entire brood cycle.

Despite these advancements, a critical bottleneck remains: the physical observation setups lack standardisation. Historically, automated studies have relied on non-standardised wooden observation hives. A primary theoretical advantage of automation should be the facilitation of higher-throughput experimental replication. However, because observation setups are complex to construct and calibrate, numerous studies remain constrained to technological proofs of concept utilising a single observation colony (e.g. Wario et al., 2015, 2017; Bozek et al., 2021; Stefanec et al., 2021; Žampachů et al., 2022; Gernat et al., 2023; Ulrich et al., 2024; Kongsilp et al., 2024). While some researchers have successfully deployed three to five hives within a single study, for example, Smith et al. (2016), Bozek et al. (2021), and Blaha et al. (2025), scaling these setups remains logistically demanding. More pressingly, the lack of cross-laboratory standardisation severely limits the transferability of analytical tools. Exact dimensions and sensor-mounting methods are rarely documented, leading to highly disparate sensor rigging. For instance, Bozek et al. (2021) mounted cameras at a 1 m distance with four-angle IR lighting, whereas Blaha et al. (2025) positioned cameras at 47 cm with direct lighting, and Wario et al. (2015) utilised custom observation cages. These vastly different optical parameters, focal lengths, and shadow profiles mean that computer vision models developed for one dataset are rarely transferable to another without extensive, resource-intensive retraining. Furthermore, these wooden setups often necessitate biological or operational compromises to overcome technical limitations. For example, to ensure camera visibility, Bozek et al. (2021) fixed the back side of the comb to a wooden surface, unnaturally constraining the bees to a single side. Operationally, long-term automated studies suffer from debris accumulation on the glass, necessitating regular cleaning (Blaha et al., 2025). Because traditional wooden frames are prone to warping and expansion from internal hive humidity, removing glass panels for maintenance is often difficult and highly disruptive to the colony. Therefore, as automated methods become increasingly central to behavioural studies, it is increasingly important for experimental setups to offer precise standardisation, operational stability, and modularity. By examining the studies in the last row of Table 1, we identified these issues thwarting reproducibility and addressed them with the open-hardware system presented herein.

**Fig. 1.**
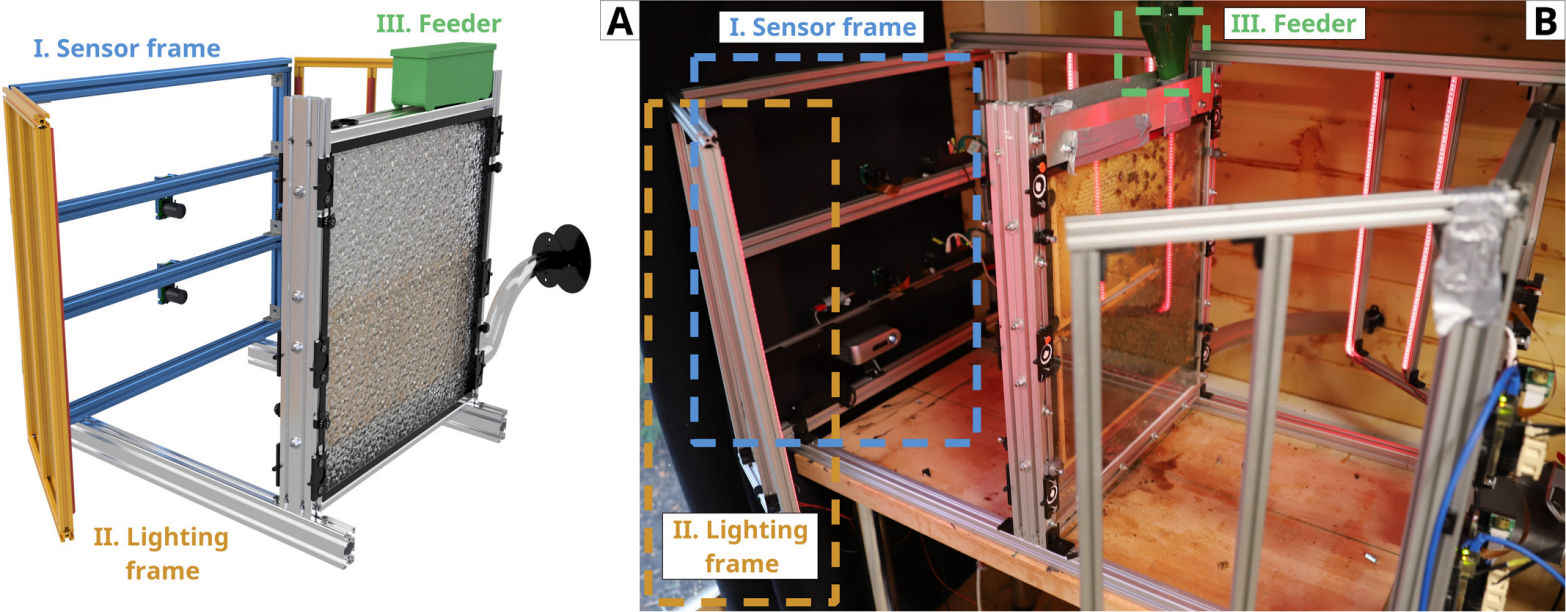
**Observation system with the hive in the middle.** (A) Rendered 3D model render. (B) Assembled hive with a live honeybee colony. Highlighted in both panels are (I) sensor frame in blue, (II) lighting frame in yellow and (III) feeder in green.

We present an observation system for honeybees that not only offers improved accuracy but is also designed for maximum reproducibility while remaining modular and adaptable to different research objectives. At the same time, the system offers benefits for the animals, as it is much easier to disinfect than previously used wooden observation hives, allowing long-term use. As a two-frame observation hive, it follows a historically established research format and uses the Zander comb size, ensuring immediate compatibility with standard beekeeping practices in Austria. However, the system is highly adaptable and can be easily modified to meet the specific requirements of different experiments and comb sizes, allowing experimental conditions to be customised while ensuring comparability between studies. The observation system consists of three primary independent components: the observation hive, the observation platform and a control computer. The overall system architecture is shown in Fig. 1. Each component is designed to be modular, standardised and versatile, allowing for easy modification, replacement and software updates to suit different experimental needs. The observation hive can be easily adapted to accommodate multiple combs or broader configurations to meet the needs of different research questions. For example, adapting the system to house standard Langstroth frames (448×232 mm) instead of the Zander frames (420×220 mm) utilised in our validation simply requires adjusting the cut lengths of the horizontal and vertical aluminium profiles during assembly. Because the structural joints, ventilation meshes, and sensor mounts are independent of these profile lengths, the hive format can be changed without requiring any redesign of the core architecture. Similarly, the observation platform can accommodate a range of sensors, from traditional cameras and thermal imagers to actuators that enable interactive experiments, providing an adaptable interface that goes beyond passive data collection. To illustrate the system's capabilities, we present a case study in which an individual honeybee queen is tracked throughout the hive, demonstrating how minimal adjustments can facilitate a broad range of experimental designs. This flexible, standardised platform allows a range of complex behavioural and interaction studies to be conducted with minimal reconfiguration, combining real-time tracking and behavioural classification capabilities, enhanced standardisation and modular adaptability.

To validate the system's suitability for honeybee research, we assessed its biocompatibility via long-term colony health monitoring over 2 months during the active foraging season and demonstrated tracking capability using the honeybee queen as a focal subject for continuous observation. This complex validation approach ensures that the system meets both technical and biological requirements and standards necessary for reliable behavioural research.

## RESULTS

The proposed hive design was evaluated to ensure fitness for the experimental setup and biocompatibility by housing a living colony for an extended period. The presented results are based on data collected at the University of Graz in Austria during May–July 2023 (two colonies, A and B) and July–August 2024 (four colonies, C–F). The observation system design is illustrated in Fig. 1, showing both the rendered 3D model (A) and the assembled hive with a live colony (B).

### Colony health assessment

Throughout the observation periods, weekly qualitative inspections were performed by professional apiarists following standard beekeeping practice. At each inspection, colonies were checked for normal foraging activity at the hive entrance; the presence of eggs, larvae, and capped brood as evidence of continuous brood rearing; and visible signs of disease or distress (e.g. deformed wings, crawling bees, discoloured or perforated brood cappings). Across all six colonies and all inspection time points, foraging activity and brood rearing were consistently observed, and no visible pathogens or signs of colony distress were detected. These qualitative observations are complemented by quantitative population data. Healthy colonies maintain relatively stable populations without significant declining trends; substantial population decline would indicate potential health issues. Population size measurements, together with their mean values and standard deviations of all six colonies, are presented in Tables 2 and 3. Colonies A–F showed low coefficients of variation (CVs), indicating relatively stable colony sizes (A: CV=0.175, B: CV=0.097, C: CV=0.045; D: CV=0.09; E: CV=0.075; F: CV=0.075). A negative trend using the Theil–Sen slope estimator was observed in both 2023 colonies (A: slope=−155.3; B: slope=−38.5). Kendall's rank correlation indicated no significant monotonic association between time and population size in either colony (A: *τ*=−0.429, *P*=0.239; B: *τ*=−0.238, *P*=0.562). The population trend in E (slope=+52.6) had the most positive population change. The monotonic increase was approaching statistical significance (*τ*=0.571, *P*=0.061). In contrast, colonies C, D, and F showed no significant monotonic trends, with weak effect sizes indicating the absence of a sustained population change (C: *τ*=−0.357, *P*=0.275; D: *τ*=0.214, *P*=0.548; F: *τ*=0.071, *P*=0.905).

**Table 2.** Population size measurements for 2023 colonies (*n*=2) over time, used to evaluate mortality and validate the biocompatibility of the hive

Date	A	B
2 May 2023	2998	−
10 May 2023	3352	2521
17 May 2023	3492	3068
22 May 2023	3225	3164
31 May 2023	2663	2974
5 June 2023	1991	2485
12 June 2023	−	2914
13 July 2023	2759	−
14 July 2023	−	2634
mean±s.d.	2926±511	2823±273

No measurement for a given day is indicated by ‘−’.

**Table 3.** Population size measurements for 2024 colonies (*n*=4) over time, used to evaluate mortality and validate the biocompatibility of the hive

Date	C	D	E	F
30 June 2024	2571	2365	2506	2456
8 July 2024	2896	2846	2874	2825
15 July 2024	2772	2994	2813	2716
22 July 2024	2609	2331	2639	2255
29 July 2024	2660	2703	3042	2588
4 August 2024	2665	2850	3072	2815
12 August 2024	2555	2889	3087	2760
19 August 2024	2562	2813	3026	2608
mean±s.d.	2661±119	2724±246	2882±217	2628±196

Comparison of population sizes across colonies using the Mann–Whitney *U* test for 2023 and the Kruskal–Wallis *H* test for 2024 showed no statistically significant differences among colonies (2023: *T*=32.0, *P*=0.383, 2024: *T*=6.439, *P*=0.092), indicating substantial overlap in population distributions. Week-to-week population differences of all colonies (A–F) revealed no significant median change in either colony, indicating that short-term fluctuations did not translate into consistent change (Wilcoxon signed-rank test; A: *T*=10.0, *P*=1.0; B: *T*=10.0, *P*=1.0; C: *T*=13.0, *P*=0.938; D: *T*=9.0, *P*=0.469; E: *T*=12.0, *P*=0.781; F: *T*=13.0, *P*=0.938).

### Queen tracking

**Fig. 2.**
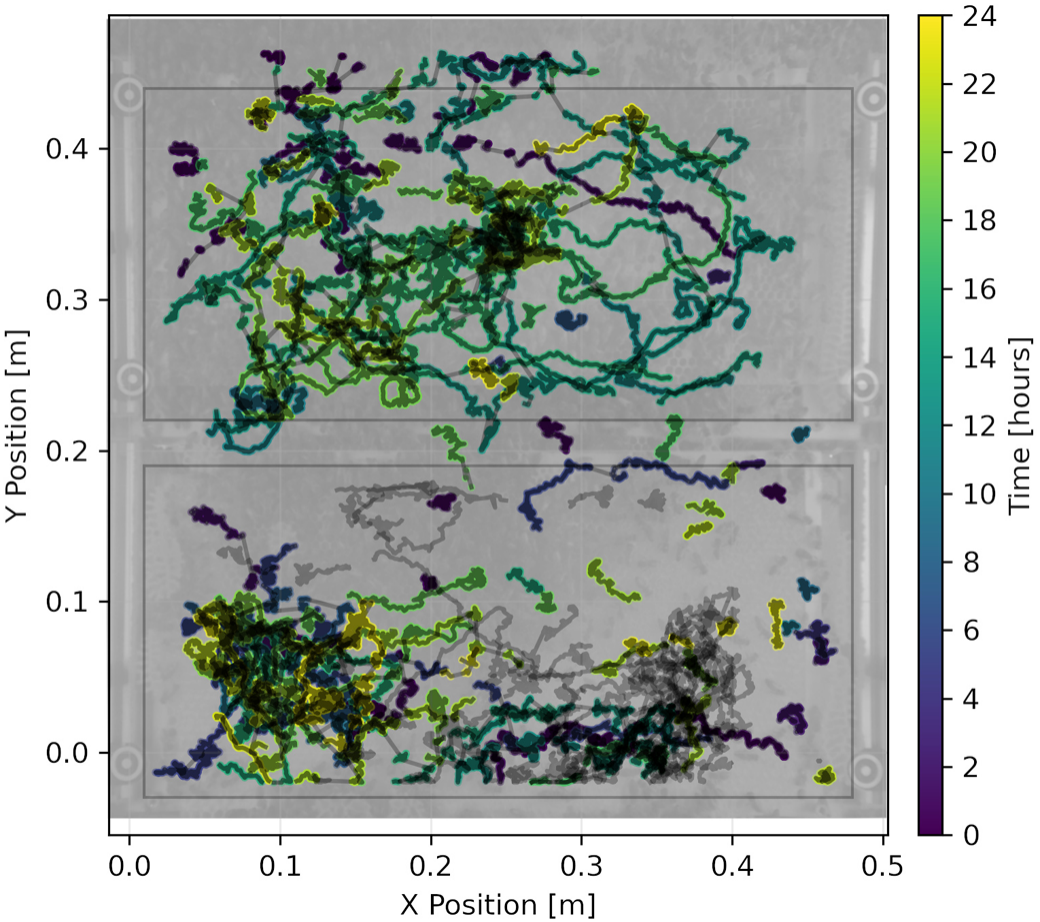
**Visualisation of a honeybee queen (*n*=1) trajectory covering one hive side recorded for a full day on 10 July 2023.** The background image is an illustrative stitched image from the experimental setup cameras. Trajectory points are coloured by the time of day at which the queen occupied each position, following the colour bar on the right [time (h)], which runs from 0 h (start of recording, dark purple) to 24 h (end of recording, yellow).

We used the WhyComb (Žampachů et al., 2022) system to continuously track the honeybee queen's position in time and obtain detailed images of the queen's immediate vicinity. It demonstrates the system's capability for focused observation within the hive. Fig. 2 shows an example of a 1-day-long queen trajectory recorded on 10 July 2023, covering one side of the hive. The background image is a stitched overview of the experimental setup, provided for spatial reference. To avoid false detections, the displayed trajectory was filtered using constraints allowing detections only in the accessible area of the hive, *x*∈[0.01, 0.48] m and *y*∈[−0.02, 0.5] m, and with detector parameters class_id≤1200 and conv_val≥5000. We split the continuous tracking into smaller sequences whenever a detection jumped by more than 3 cm, and we kept sequences of at least 20 detections. The resulting path is intended as an illustrative representation of continuous tracking rather than a detailed analysis of movement dynamics, which were presented by Blaha et al. (2025). The tracking results demonstrate that the system can reliably follow the queen over extended periods and across a substantial portion of the comb surface. As reported by Žampachů et al. (2022), when analysing the images from the same system, the mean localisation error was 4.3 pixels, with a detection precision of 0.987 and recall of 1.000. Apart from the provided tracking module, other computer vision modules can extend the processing pipeline to detect worker bees or analyse comb, thanks to the standardised robot operating system (ROS) data interface as in Ulrich et al. (2024).

## DISCUSSION

Here, we present a comprehensive and standardised observation-hive design that addresses the need for greater precision arising from the growing number of studies using automated observations (e.g. Kongsilp et al., 2024; Gernat et al., 2023; Bozek et al., 2021; Boenisch et al., 2018) in honeybee behavioural research. The transition to more automated approaches demands more precise observation setups and careful calibration to ensure data accuracy, especially over extended periods. While traditional wooden observation hives have been used for centuries in numerous scientific publications (see also Table 1), our approach aims to introduce a high-precision, highly durable, yet modular observation-hive system based on readily available, standardised components. Combined with automated data collection methods, this hive not only accommodates flexible research needs but also helps meet higher accuracy requirements and supports continuous data collection, overcoming the typical limitations of human observation. The presented hive design was already used to support the findings of Ulrich et al. (2024) and Janota et al. (2024), where a honeybee colony was observed by cooperating robots. To support future research, we provide 3D computer-aided design models, part lists, and detailed assembly instructions in Figs S1–S4.

Compared to conventional wooden hives and other fabricated alternatives such as moulded plastic or acrylic, the aluminium construction provides several distinct advantages:

- Improved data collection. Designed to integrate with automated data collection systems to support the 24/7 data collection that is increasingly essential for behavioural research.
- Modularity. The system uses standard T-slot aluminium extrusions to which sensors and hardware can be attached and repositioned as required. This non-destructive adaptability enables optical parameters to be fine-tuned or the setup to be completely reconfigured, without drilling required by wooden or moulded plastic hives, depending on the research question.
- Higher levels of accuracy and repeatability. Precision-engineered construction ensures consistent dimensions and tight tolerances, improving reproducibility in experimental setups and across studies.
- Hygiene. The observation hive is fully washable and easier to clean and disinfect after infections, reducing the risk of pathogen persistence.
- Durability. Compared to wooden hives, which are prone to warping, rotting, and limited lifespan, aluminium hives last significantly longer and are fully recyclable.
- Price. The total cost (€135) lowers the entry barrier and allows more aluminium hives than the wooden hives (€260; https://bienen-janisch.at/produkt/schaukasten-fuer-za-vollzarge/). We compare only the price of the hive, as the additional frames with sensors and lighting would need to be considered in both cases. Also, their prices vary depending on the intended experimental setup.

The engineering advantages of this system enable several avenues of basic and applied research that are currently constrained by traditional setups. First, the low manufacturing cost and open-source design lower the barrier to higher throughput, multi-colony replication, allowing the scale up of setups to achieve the statistical power required for assessing environmental stressors across multiple colonies simultaneously. Second, the precise standardisation facilitates multi-site, comparative studies, allowing researchers to reliably compare honeybee behaviours across different geographical locations, climates, or local environmental conditions. Because the physical dimensions and optical parameters can be fixed by the hardware design, different laboratories can share and directly apply the exact same computer vision models across these varying conditions without resource-intensive local retraining. Finally, because the aluminium frame is fully washable and sterilisable, unlike wood, which absorbs moisture and pathogens, the system permits long-term toxicological and pathogen transmission studies where preventing cross-contamination between experimental trials is paramount.

We tested the system over an extended period of time and observed robust colony health, indicating successful acclimation to the hardware. As detailed in our population assessments (Tables 2 and 3), the colonies maintained stable or growing population sizes throughout the active foraging season. Adult population size can be interpreted as a proxy indicator of colony health during the active foraging season, on the following basis. Summer worker bees have a lifespan of approximately 25–35 days (Winston, 1991; Rueppell et al., 2007), so the adult population observed at any time point reflects the ongoing balance between forager mortality and successful brood rearing. A colony with a failing queen, insufficient brood care, or severe pathogen load would show a population decline within 2–4 weeks as dying workers fail to be replaced. Stable or increasing population trajectories during summer therefore imply continuous successful brood rearing and an absence of acute colony-level stressors. This indicator is appropriate for the summer active period only; winter population dynamics follow a different pattern due to the extended lifespan of workers and cannot be interpreted analogously. The population size is a coarse indicator that will not resolve subclinical pathogen loads or sublethal physiological stress, but it is sufficient for the purpose of hardware biocompatibility validation, and it is complemented by the weekly qualitative inspections performed by a professional apiarist, who continually verified normal foraging activities, active brood establishment, and an absence of disease.

The fundamental contribution of this work remains its low-cost, open-source framework, which leverages the high-precision manufacturing tolerances of standard aluminium extrusions to ensure exact structural and optical reproducibility. This framework provides a standardised platform that meets the growing demand for reproducible, automated observation in honeybee research. Whereas existing automated setups are typically purpose-built around a single research question, the observation hive presented here accommodates a broad range of experimental settings, from passive behavioural recording and thermal mapping to active interaction studies with vibrational, thermal, or robotic stimuli, simply by exchanging the sensor or actuator mounted on the observation frame.

## MATERIALS AND METHODS

The presented observation system consists of three main independent parts: (1) the observation hive housing the honeybees behind the glass; (2) the observation frame with the sensors (e.g. cameras); and (3) the control computer responsible for data processing, storage, and uploading; see Fig. 1A for the rendered model and Fig. 1B for a real hive with a living colony. The entire system is based on standard extruded aluminium profiles and 3D-printed parts, which facilitate rapid, cost-effective prototyping and make it more adaptable to different experimental setups. This design choice also ensures the system's easy reproducibility, availability, and modularity. We present an example experimental setup used for tracking a single individual, the honeybee queen. The observation frame was used from both sides to hold cameras, one for each comb side, and near-IR (NIR) illumination, so as not to disturb the colony. The cameras were operated by dedicated single-board computers, and the data were further streamed, processed and stored by a central master computer. We provide a bill of materials in Tables S1–S5 and a basic assembly schema in Fig. S1. The 3D model, 3D-printable parts and software are provided in the Zenodo repository (https://zenodo.org/records/20641893).

### Mechanical design

The hive is designed as a drop-in replacement for traditional two-frame wooden observation beehives. It is constructed primarily from standard aluminium extrusions (Al profiles), which are widely used in laboratory and industrial applications due to their standardised design, ease of machining and simple assembly. In addition, the aluminium profiles make it easy to fit additional equipment and allow the hive to be customised for different experimental setups or comb sizes. The frame material is also easy to clean in a washing machine or pressure washer and can be disinfected if necessary.

The main frame of the hive has external dimensions of 545×620 mm, excluding the support stand. The main frame can therefore be constructed using 2.5 m of 3060 aluminium profile. The top and bottom of the frame have five 32 mm holes (two on the top and three on the bottom), each with a 3D-printed mesh to facilitate ventilation and feeding. A separate feeder box can be attached as in Fig. 1, III. One side of the frame contains a 32 mm hole with a 3D-printed passthrough that connects to a transparent PVC tube with an outer/inner diameter of 50/40 mm. This tube provides a passageway for bees to enter and exit the hive freely. In addition, four 210 mm sections of 2040 aluminium profile are attached to the inside of the frame to support full 1/1 Zander wooden combs. We have chosen full 1/1 Zander combs for their compatibility with local standard beekeeping practices. Glass panels measuring 483×509×4 mm are secured on each side by four 3D-printed tabs with thumb screws, allowing easy removal while wearing gloves. To ensure complete biocompatibility and prevent any potential leaching from the metal frame, we have also designed an optional, 3D-printable polymer lining for all internal surfaces. An analysis of hive products informed this design decision and is provided in Table S6. The corresponding 3D models are included in the supplementary information. The hive is mounted in the centre of two 3030 profile rails (minimum length 1100 mm per setup), which ensures the structural rigidity of the system and ease of attaching the observation frame. Fig. S1 shows the assembly schema, and it includes a self-contained part list and detailed measurements to exactly replicate the hive.

The observation hive can be upgraded to an automated long-term focus observation system by incorporating cameras and lighting setups mounted on two observation frames, positioned on each side of the hive. This extended configuration involves attaching cameras and lights to frames constructed from 3030 or 2020 aluminium profiles, with the profile type selected based on the specific requirements of the mounted equipment. The observation frame consists of a sensor frame (Fig. 1, I) and a lighting frame (Fig. 1, II). The sensor frame is constructed from horizontal profiles aligned with the centres of the honeycombs, allowing cameras to be mounted using 3D-printed brackets. The lighting frame, connected to the sensor frame by hinges with stop blocks, provides easy access for maintenance or glass replacement, while allowing precise positioning of the lights to minimise reflections. The lighting frame consists of vertical aluminium profiles to which the LED strips are attached using built-in adhesive. These profiles also act as cooling rails for the LEDs, improving thermal management. The whole setup requires less than 10 m of 2020, 1 m of 2040, 2.5 m of 3060, and 10 m of 3030 of aluminium profiles, which are the recommended lengths for ordering. We list the mechanical, 3D-printed, and electronic components used for the hive unit (Tables S1 and S2) and the observation frame (Tables S3 and S4), as well as the associated observation system processing hardware (Table S5). For each part, we include quantities, material specifications, approximate prices (where available), and relevant notes explaining their function or purpose within the system. These tables are intended to assist in replication or modification. Note that the prices are just estimates and may vary depending on suppliers.

### Tracking system

To support the long-term observations and experiments enabled by the presented hive, we have developed an open-source data processing and management system. The system is based on ROS, a software framework widely used not only in robotics (Koubaa, 2017), but also in behavioural research (Ulrich et al., 2024), agriculture (Shamshiri et al., 2018; Tsolakis et al., 2019), biomonitoring (Resende et al., 2021), and other challenging environments (Macenski et al., 2022). ROS provides a multi-language, abstracted communication framework for programs and computers, enabling platform-independent development of computing systems. The programs and communication relationships in the ROS framework form a graph, where nodes represent running programs and edges represent data connections between them. By abstracting and standardising communication with human-readable data stream definitions, data can be automatically stored and processed in the long term. To further support reproducibility, users should calibrate camera parameters using a standardised chessboard pattern (Zhang, 2002) and set focus and exposure settings to minimise motion blur for accurate imaging.

Having standardised camera images, it is possible to process them by computer vision methods for bee detection and tracking. This enables tracking of single individuals as well as tracking of multiple bees. There are methods (e.g. Wario et al., 2015; Crall et al., 2015; Žampachů et al., 2022; Gernat et al., 2023) that detect specialised markers with black-and-white patterns to estimate the bees' position. Apart from the position, the markers may provide unique identification and orientation estimation. To avoid the laborious manual marking of each bee, markerless tracking methods (Bozek et al., 2021; Kongsilp et al., 2024) use neural networks to detect and track each bee. However, this method consumes more computational resources than geometric approaches based on marker detection.

Our system, WhyComb (Žampachů et al., 2022), is tailored for continuous focused observation and tracking of a single individual, the honeybee queen. Tracking multiple worker bees with the system is possible, but it would require carefully tagging each bee. There would also be a risk of losing the tags or the bees during their foraging flights. Thus, for system validation, we track only the queen. WhyComb system derivatives are commonly used to track swarms of mobile robots with up to ten individuals (Arvin et al., 2018). However, the markers in these scenarios are larger, which, in our case, would require doubling the camera resolution, as the workers being physically smaller could not fit a sufficiently big marker on their thoraxes. The WhyComb system derivatives are aimed at detection efficiency rather than identification of different individuals. Therefore, in scenarios, where one would need to track a high number of individuals, one would use one of the systems mentioned before (Wario et al., 2015; Crall et al., 2015; Gernat et al., 2023; Bozek et al., 2021; Kongsilp et al., 2024).

In our setup, we used one camera per comb side with a resolution of 4056×3040 pixels at 10 Hz, for a total of four cameras, to achieve sufficient detail. So the system consists of four identical processing pipeline instances that evaluate the comb images, as depicted in Fig. S2, and the overall hive system then decides on the tracking information and prepares the data for storage. The pipeline starts with a camera node that reads images from the connected camera. The captured image is passed to the WhyComb marker-tracking nodes, which provide a position estimate in the hive-defined coordinate system and a smaller cropped image of the immediate queen vicinity to reduce bandwidth and storage requirements. The camera image, cropped image and position estimation of all four pipelines are fed into the hive's collector node, which decides and outputs which tracking pipeline should be used in situations where the queen moves across combs or even changes hive sides. The collector node's output is then stored as a rosbag file, a native ROS data structure that preserves the system's timestamped dataflow. Along with the data pipeline, our system includes additional monitoring and management tools that automatically upload recorded data to network storage or power cycle in the event of failure (see Figs S3 and S4).

The aforementioned WhyComb marker is a tailored WhyCode (Ulrich et al., 2023) fiducial marker tracker for honeybee hives to overcome occlusions by other bees' antennae that often occur during interactions. The marker is attached to the dorsal thorax of the queen and is similar in shape to the widely used beekeeping markers for queen identification. The marker consists of two black-and-white concentric circles and has a diameter of 3 mm. For durability, it is printed on Xerox NeverTear paper and coated with Belton acrylic varnish, which enhances the longevity of both the paper substrate and the printed pattern. All the used electrical and electronic components are provided in Table S5.

### Biological validation

To assess the biological suitability of our aluminium-framed observation hive, we monitored colony development during May–July 2023 (colonies A and B) and July–August 2024 (colonies C–F) to evaluate potential impacts on colony health and productivity. Six honeybee colonies (A–F) were placed in the observation hive and monitored for changes in population size, brood production, and general vigour to assess their well-being. To assess the colony health qualitatively throughout the observation period, professional animal caretakers conducted weekly routine inspections following standard beekeeping practices, such as the Liebefeld method (Imdorf et al., 1987) and the COLOSS BEEBOOK protocols (Human et al., 2013). During these qualitative inspections, normal foraging behaviour, continuous brood rearing, and the absence of visible pathogens or distress were checked. We note that these qualitative checks were not designed to quantify brood survival rates or establishment dynamics but rather to confirm the absence of overt colony health problems during the hardware validation period. Colony size was estimated from images recorded by the observation system. A human annotator counted bees in the images from 2023, while images from 2024 were automatically obtained and processed by a convolutional neural network following Ulrich et al. (2024). As the automatic method did not count partially visible bees, we scaled the automated population counts by the ratio (1.418) of human-annotated to automated counts. The ratio was derived by manually annotating images from 3 days (30 June, 29 July, and 19 August) of colony C and comparing the counts to the estimated population counts obtained from the same data using the automated method. The difference could be reduced by restricting bees from crawling over each other by a smaller distance between glass panels and wax combs or by using artificial combs, which can have a more even surface to prevent local valleys where the bees can gather.

To characterise temporal population dynamics across six colonies (A–F), we tested whether population sizes exhibited significant monotonic trends over time and whether inter-colony population distributions differed significantly. Given the small sample sizes, non-parametric statistical approaches were employed to ensure robust inference, as parametric tests require larger samples for reliable validity. Temporal trends were quantified using the Theil–Sen estimator (slope) and Kendall's *τ* (monotonic association), with significance determined via *P*-values. Colony stability was evaluated through CVs, while inter-colony comparisons utilised the Mann–Whitney *U* test (2023) and Kruskal–Wallis *H* test (2024). Short-term week-to-week fluctuations were analysed with the Wilcoxon signed-rank test. All analyses were conducted at *α*=0.05.

### Biological material and environmental conditions

The experiments were conducted using *Apis mellifera carnica* Pollmann, a honeybee strain endemic to Styria and Carinthia, Austria. The colonies were commercially sourced from the Styrian beekeeping centre in Graz and maintained by professional beekeepers at the University of Graz. The age and sex demographics of the colonies were not artificially manipulated for the study. Each colony followed a natural composition, containing a queen and workers alongside a small number of drones. All procedures adhered to the Austrian Animal Experiments Act (TVG 2012, 1. Abs., §1) and internal institutional ethics standards; notably, the TVG 2012 exempts insect research from formal ethical approval requirements. The bees were provided unrestricted foraging access to the surrounding urban campus of the University of Graz via a transparent PVC flight tunnel. To preserve natural behaviour and ensure observations were conducted in the bees' perceived darkness, the hive was illuminated using 780 nm NIR LED strips. This wavelength sits outside the visual spectrum of honeybees, allowing for 24/7 monitoring without light-induced behavioural disruption. The LED strips were mounted to the lighting frame outside the hive, which also served as a cooling rail; consequently, the thermal input from the illumination was negligible and did not significantly affect the hive's internal homeostasis. Prior to the commencement of data collection, the queen bee was marked for automated tracking using a fiducial marker. The marker was secured to the dorsal thorax using a shellac-based adhesive to ensure biocompatibility and long-term adhesion.

### Use of artificial intelligence tools

During the preparation of this work, the authors used Grammarly and DeepL to improve the spelling, grammar, clarity, and overall readability of the manuscript. After using these tools, the authors carefully reviewed and edited the text as needed and take full responsibility for the final content of the publication.

## Supplementary Material



10.1242/biolopen.062523_sup1 Supplementary information
