# Supplementary material for: An open-source high-precision hive for long-term honeybee observation and research
Source: Biol Open. 2026 Jun 30;15(6):bio062523. doi: 10.1242/bio.062523 (PMC13382704; doi:10.1242/bio.062523)
Supplement: Supplementary information [file biolopen-15-062523-s1.pdf]

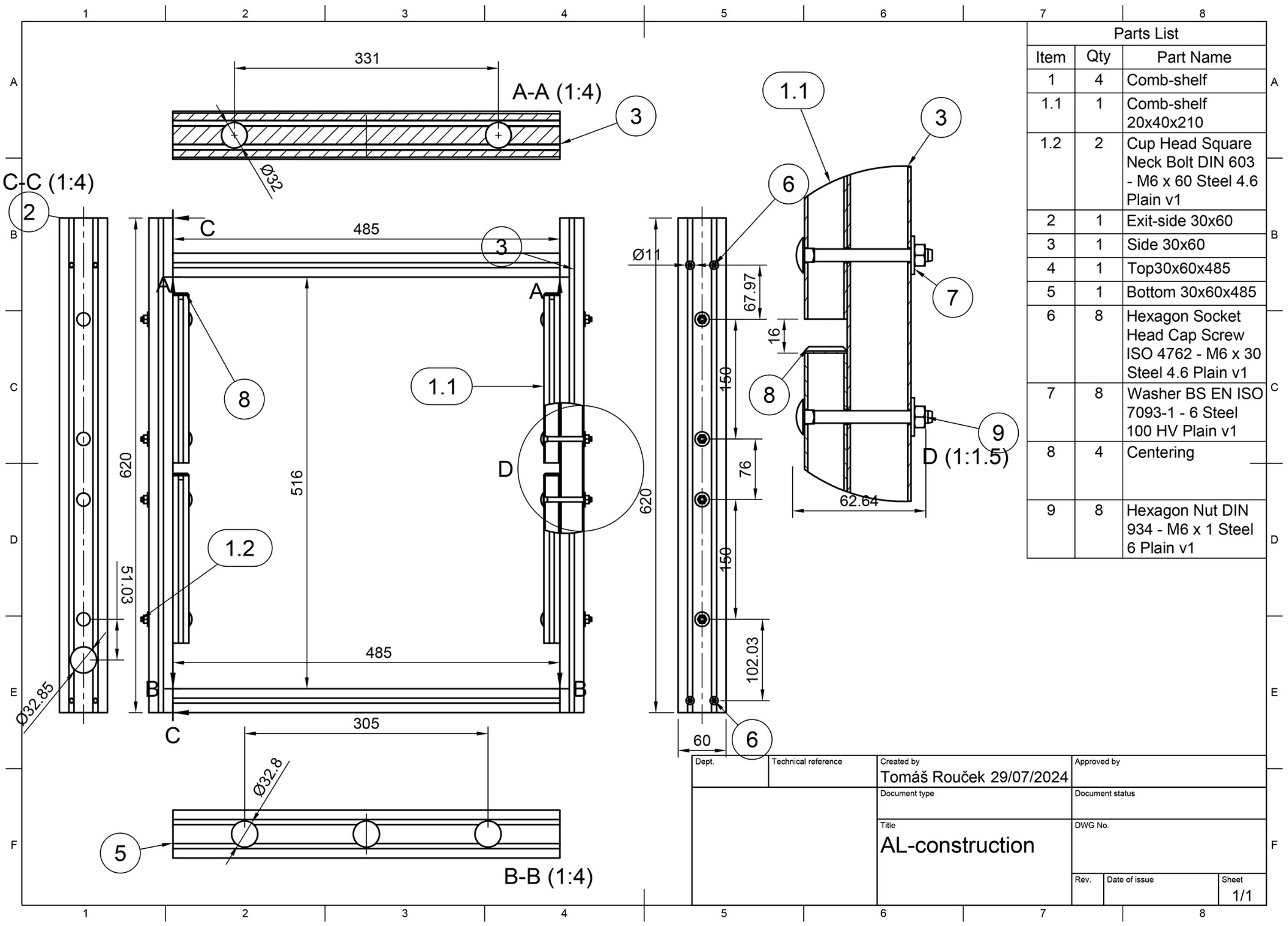

Fig. S1. Observation hive construction schematics with part list.

Biology Open • Supplementary information

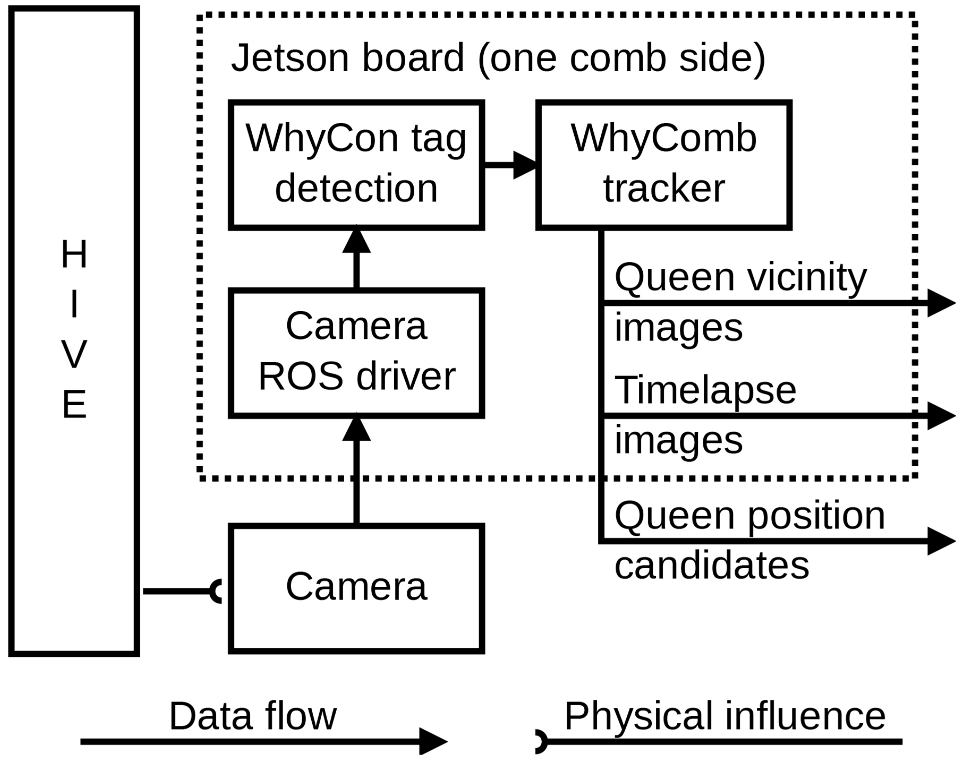

Fig. S2. Vision module nodes schematics running on a Jetson Nano computer. WhyComb outputs are sent to the Master computer. Nodes auto-start on boot.

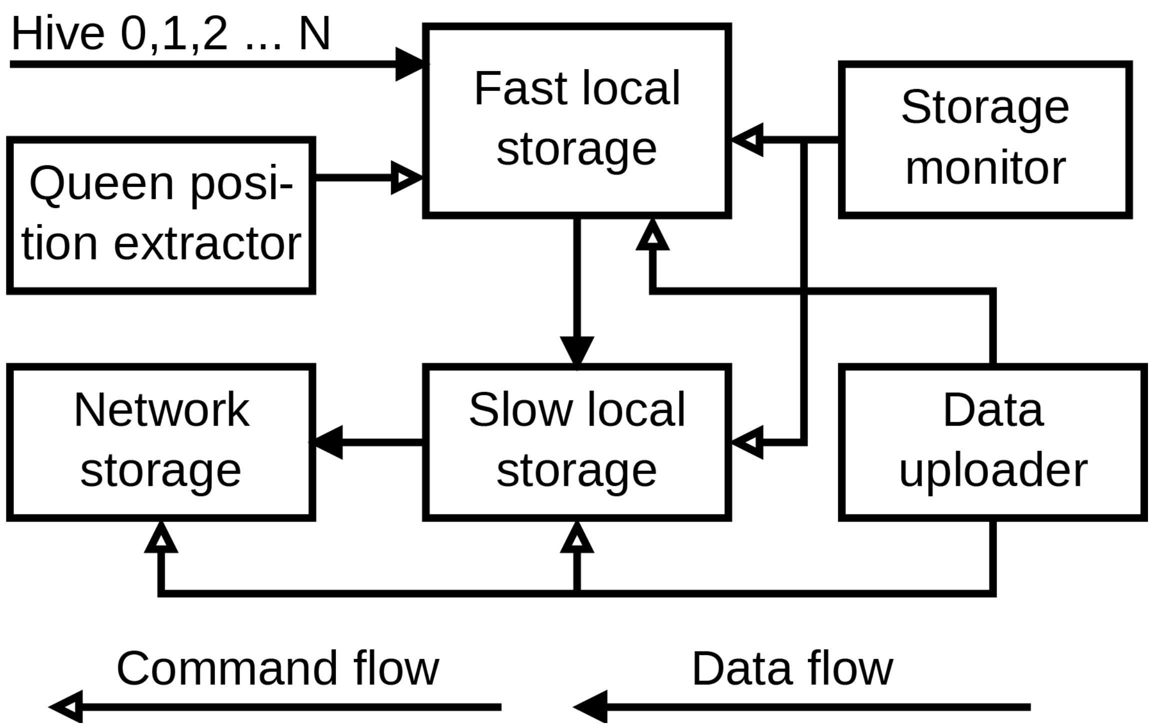

Fig. S3. Data management modules schematics for hierarchical (local/remote) storage running on the Master computer. Data are stored as ROSbags with metadata (messages types/counts). Modules auto-start on boot.

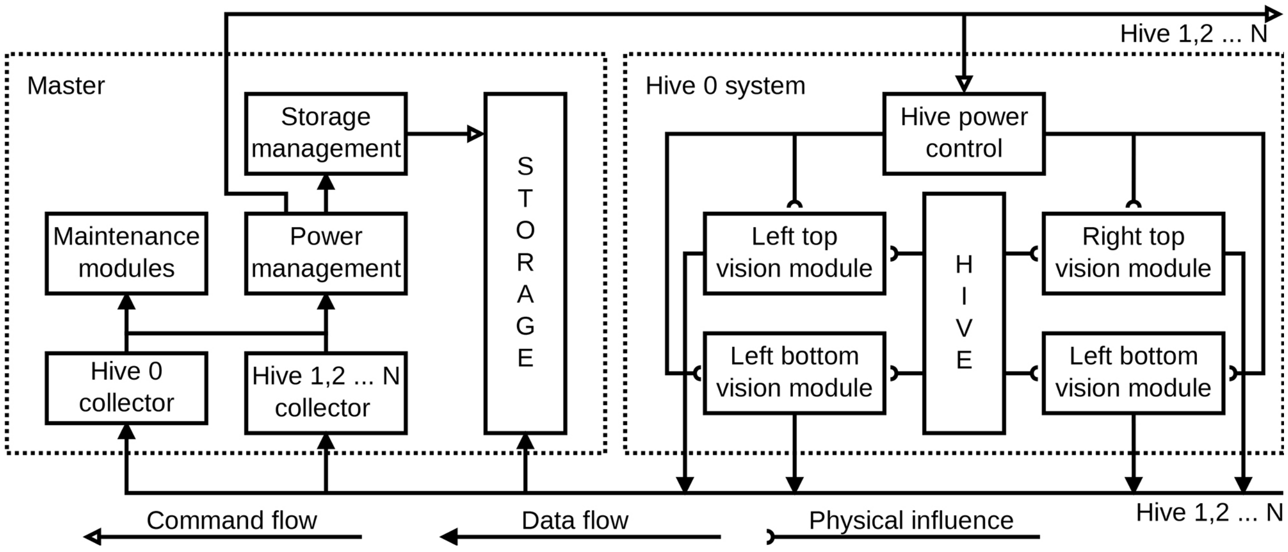

Fig. S4. Software structure of multi-hive observation system. Collector nodes aggregate data per system and identify queen images, positions, and timelapse images from vision modules for storage. They monitor data flows for automatic resets and control power for remote restarts or shutdowns.

Biology Open • Supplementary information

Table S1. Al hive - Mechanical components. The whole hive can be placed on longer feet by extending the sides of the construction.

Item	No.	Price / pc [€]	Price / total [€]	Notes
**Aluminium profiles**			**83.8**	
Item 5 (2040) 210 mm	4	2.1	8.4	Comb + glass mounting
Item 6 (3030) 1100 mm	2	11.0	22.0	Rails
Item 6 (3060) 620 mm	2	12.4	24.8	Sides; one side flat
Item 6 (3060) 485 mm	2	9.7	19.4	Top, bottom; one side flat
Item 6 L bracket	4	2.3	9.2	
**Fasteners**			**18.56**	
DIN 603 M6x60	8	0.15	1.20	
DIN 7093 - 6	8	0.02	0.16	
DIN 7991 M5x12	24	0.03	0.72	Marker mounts
DIN 7991 M5x16	4	0.04	0.16	Tube mount
DIN 7991 M6x12	8	0.05	0.40	For L brackets
DIN 934 M6	8	0.01	0.08	
ISO 4032 M4	12	0.03	0.36	
ISO 4762 M4x25	12	0.03	0.36	Alternatively DIN 464 M4x16
ISO 4762 M6x30	8	0.09	0.72	
T-slot nut Item 6 - M4	12	0.30	3.60	
T-slot nut Item 6 - M5	28	0.30	8.40	Markers + tube mount
T-slot nut Item 6 - M6	8	0.30	2.40	For L brackets
**Others**			**42**	
Comb (Zander size)	2	3	6	
Glass 483x509x4 mm	2	13	26	
Tube 50/40 mm out/in ⌀ [m]	1	10	10	Escape path for bees

Biology Open • Supplementary information

Table S2. Al hive - 3D-Printed components.

Item	No.	Material	Notes
Ramp	1	PLA	Support bees to crawl out
Tube mount	1	PLA	
Glass holding peg	8	PETG	
Glass holding screw	8	PETG	Alternatively DIN 464 M4x16
Marker mount	12	PETG	Outline hive coordinate system
Ventilation plug	5	PLA	
Feeding plug	2	PLA	
Feeder with cap	1	PLA	Alternatively insert a bottle through the feeding plug
Inner coating	1	PLA	Recommended if single-flat-side profiles are unavailable and to reduce the aluminium exposure

Table S3. Observation frame - Mechanical components. Quantities are listed for the whole observation setup, so two observation frames. Camera frame can utilize Item 5 (2020) for cost reduction. Light frame can utilize Item 6 (3030) for parts standardization. Fasteners must match aluminium profile changes.

Item	No.	Price / pc [€]	Price / total [€]	Notes
**Aluminium profiles**			**141.6**	
Item 5 (2020) 330 mm	8	1.7	13.6	Light frame
Item 5 (2020) 550 mm	12	2.8	33.6	Light frame
Item 6 (3030) 550 mm	4	5.5	22.0	Camera frame
Item 6 (3030) 640 mm	4	6.4	25.6	Camera frame
Item 6 (3030) 710 mm	4	7.1	28.4	Camera frame
Item 6 L bracket	8	2.3	18.4	Camera frame
**Fasteners**			**18.6**	
DIN 7991 M5x10	32	0.02	0.64	
DIN 7991 M6x12	16	0.03	0.48	For L brackets
ISO 7380 M5x16	8	0.06	0.48	
ISO 7380 M6x20	24	0.09	2.16	
T-slot nut Item 5 - M5	8	0.30	2.40	
T-slot nut Item 6 - M5	24	0.30	7.20	
T-slot nut Item 6 - M6	16	0.30	4.80	For L brackets

Biology Open • Supplementary information

Table S4. Observation frame - 3D-Printed components. Quantities are listed for the whole observation setup, so two observation frames. Mount points need to be adjusted if using different aluminium profiles.

Item	No.	Material	Notes
Camera mount	4	PETG	Holds camera on a profile
Camera light sensor cap	4	PETG	Force IR vision
Jetson mount	4	PETG	Integrated for the MIPI-CSI extension
Light angled brackets	8	PETG	Join light frame to camera frame

Table S5. Observation system - Electronic and electrical components. Quantities are listed for the whole observation setup. The Jetson Nano, Master PC, and related components are optional, as the Robot Operating System abstraction enables flexible hardware and sensor selection.

Item	No.	Price / pc [€]	Price / total [€]	Notes
Jetson Nano	4	100	400	For each camera
USB stick 32GB	4	10	40	Bootdevice
Raspberry Pi power supply USB-C	4	8	32	5.1 V / 3.0 A
TP-Link Tapo P100	2	9	18	Remote power control
ArduCam HQ IR-CUT IMX477 camera	4	60	240	Prod. No.: SKU-B0274 Rev. D1
ArduCam CSI-to-HDMI extension	4	14	56	Prod. No.: SKU-B0091
Cable HDMI<->HDMI 1 m	4	2	8	
LED strip 780nm 24V [m]	8	80	640	
LED power supply 24V	1	60	60	
Two-core cable ⌀ > 0.25 mm^2 [m]	10	0.75	7.5	
Ethernet switch 1Gbps, 5 ports	1	30	30	
Master PC	1	-	-	Control Jetsons and process the data. Configuration can vary.
Ethernet cable	5	4	20	Jetsons, PC and switch

Biology Open • Supplementary information

Table S6. Mean aluminium content mg kg^{-1} in honey from observation (G-I) and control (J-L) hives.

Sample	Aluminium content
Colony G	7.620 ± 1.910
Colony H	0.656 ± 0.328
Colony I	Below limit of quantification
Colony J	0.450 ± 0.225
Colony K	0.735 ± 0.184
Colony L	2.330 ± 0.582
