# Supplementary material for: An open-source high-precision hive for long-term honeybee observation and research
Source: Biol Open. 2026 Jun 30;15(6):bio062523. doi: 10.1242/bio.062523 (PMC13382704; doi:10.1242/bio.062523)
Supplement: Supplementary file 1 [file biolopen-15-062523_review_history.pdf]

An Open-Source High-precision Hive for Long-Term Honeybee Observation and Research

Jiří Ulrich, Tomáš Rouček, Laurenz Alexander Fedotoff, Harald Pascher, Zdeněk Rozsypálek, Thomas Schmickl, Tomáš Krajník and Martin Stefanec
10.1242/bio.062523

Editor: Kendra J. Greenlee

Review timeline

Original submission:	2 March 2026
Editorial decision:	11 March 2026
First revision received:	13 April 2026
Editorial decision:	28 April 2026
Second revision received:	14 May 2026
Accepted:	19 May 2026

Original submission

First decision letter

MS ID#: bio.062523

MS Title: An Open-Source High-precision Hive for Long-Term Honeybee Observation and Research

Authors: Jiří Ulrich, Martin Stefanec, Tomáš Rouček, Laurenz Alexander Fedotoff, Harald Pascher, Zdeněk Rozsypálek, Thomas Schmickl and Tomáš Krajník

I have now reached a decision on the above manuscript.

The reviewer reports are shown at the bottom of this email.

Both reviewers found the paper to be interesting and timely. However they raised a number of substantial criticisms that prevent me from accepting the paper at this stage. In particular, both reviewers noted that descriptions of other methods were too vague to allow readers to understand how the new system improves on the existing technology. They also pointed out concerns with reporting of statistical tests, some data that is not reported in the results section and methods that are missing or lacking detail. If you think that you can deal satisfactorily with the criticisms on revision, I would be pleased to see a revised manuscript.

At this stage, we also ask you to ensure your manuscript complies with our formatting guidelines. Provided you are able to fully address the referees' comments, we are positive about publication of your paper (we accept over 95% of revision submissions) and therefore hope you won't mind any extra work involved in reformatting your manuscript at this point.

Please upload both a 'clean' version of your Word file, along with a highlighted version clearly showing where you have made changes in the revised manuscript. Please avoid using 'Track changes' in Word files as these are lost in PDF conversion.

I should be grateful if you would also provide a point-by-point response detailing how you have dealt with the points raised by the reviewers in the 'Response to Reviewers' box. Please attend to

© 2026. Published by The Company of Biologists under the terms of the Creative Commons Attribution License (https://creativecommons.org/licenses/by/4.0/).

all of the reviewers' comments. If you do not agree with any of their criticisms or suggestions please explain clearly why this is so.

Reviewer 1

Comments for the author

This manuscript aims at reporting an open-access technology for tracking bees in an observation hive. This is certainly of interest for the field and the methods are easy-to-follow and sufficiently described to be re-used by other labs. I particularly appreciated the effort made to evaluate the budget. I therefore have only a few recommendations to improve the readiness of the need for a standardized methodology as claimed by the authors but also its potential applications. 1. Thank you for the table 1 listing the main bibliography of the topic. However, it requires quite an effort to make sense of it. Would it be possible to add information about e.g. the main focus/question of the study, the level of technology involved and in particular for the last part, most relevant for the current paper, highlighting the differences between the different setup ? 2. Could you better illustrate the diversity of methods used in different studies and why this may have an impact on results ? 3. I understand only at the end of the discussion the purpose of table 2 (counting bees) in particular as you seemed not have used your automatic tracking system to this end. If I am correct, this aimed at evaluating mortality in your observation hive ? This should be better explained in the table caption and the results section. 4. As it stands, your software can only track the queen as far as I understood. Could you comment on potential add-ons, maybe with other tracking systems from different studies that may exist as open-source ? For example, would it be possible to follow individual workers with your labeling systems ? How many in parallel ? Would it be possible to overcome the issue of bees overlapping at it impacted the automatic counting apparently ? Finally, would the software need to be trained with labeled frames to be able to track the queen, if yes could you quantity and explicit the necessary work ?

Reviewer 2

Comments for the author

This is an interesting and potentially valuable methods paper. The overall concept is strong, and the manuscript is framed around an important problem for the field: the need for more standardised, reproducible platforms for automated honeybee observation. The emphasis on open hardware, compatibility with existing beekeeping practice, and the goal of improving comparability across studies are all compelling. I also think the broader motivation is timely, given the increasing use of computer vision and automated tracking in behavioural and applied honeybee research. However, I think there are a few key issues with the manuscript that need to be addressed.

First, the novelty of this system relative to existing systems, especially for automated tracking, is not well described. For example, the introduction discusses identifying and addressing shortcomings of existing systems, including developing a system that is "modular and adaptable." However, the description of these other systems is vague, so it is hard to assess what the specific existing shortcomings are or how the system presented here addresses them. In addition, it is not entirely clear how the challenges of modularity and adaptability are addressed in practice. What is the range of research applications for which this system could potentially be used, and what makes it more adaptable to a wider range of applications than other automated systems? Is this primarily because it is open source, and thus modifiable? Are there other practical barriers that limit reproducibility and standardisation? Relatedly, some of the key terms used here, such as "precision" and "reproducibility," are a bit vague, and the manuscript would benefit from clarifying what the authors mean by these terms in this context.

Second, the example validation dataset is not sufficiently described. There are several issues with the reporting of statistics and results, as noted below. More importantly, many key details, including the analyses, validation procedures, performance metrics, and biological outcome measures, are not described clearly enough in the current Results section or previewed in the Introduction. This makes it difficult to assess what was measured, how it was validated, and how strongly the presented data support the manuscript's conclusions. While some of these details can

© 2026. Published by The Company of Biologists under the terms of the Creative Commons Attribution License (https://creativecommons.org/licenses/by/4.0/).

be found in the Materials and Methods, at least high-level descriptions should be included earlier in the manuscript to provide readers with context for understanding the capabilities of the system.

Finally, the potential applications of this system are not well described. In particular, what types of research, whether basic or applied, would be enabled by this system that are not yet possible? Providing a few concrete examples would help readers understand the benefits of the system and its potential for broader adoption.

See detailed comments on these and other points below.

Specific comments

(FYI, the PDF had multiple line-numbering schemes; I have used the smaller numbers visible in my PDF version here. Also, I focused these comments on the first document presented in the PDF, but there appeared to be some inconsistencies between this version and the tracked-changes version also included.)

L15-197: Important to note here that honeybee populations are not declining per se; there are high annual losses, but total colony numbers are generally stable or potentially increasing.

L98: The use of "rigour" here seems a bit vague. These tools could certainly be used for experiments we would not call rigorous, and traditional approaches can also be highly rigorous. Rephrase?

L154: Does "comparative" here mean multiple species, or comparison across conditions, experimental setups, etc.? Please expand.

L156-167+: Table 1 does not specify what these limitations are, so it would be helpful to articulate them, as this is central to describing the novelty of the system relative to other automated honeybee tracking systems. In particular, the authors emphasise the system as being "modular and adaptable," but it is not clear to what extent this is true, or not true, for existing systems, or how the approach here differs.

L165: "uses the widely used ..." is repetitive.

L209-211: A bit vague. Does the current setup work for, for example, Langstroth hives? If not, what would need to be changed?

L255: The connection between this estimate of the number of bees in a hive, presumably from tracked data, though that is not actually very clear from the text, and colony health is not very clear. Is it also possible to disentangle changes driven by tracking performance, for example tag loss or detectability effects, from true population effects here?

L259-260: Is there a statistical test of this difference? Similar comments apply to other comparisons below.

L260: Similarly, is there a statistical test for what is presumably a linear model?

L298-299: Test statistics should be reported in addition to p-values.

L302-305: It is not clear what "qualitative tracking" means here. The WhyComb system needs to be described in more detail here, or at least its outputs and capabilities as relevant for interpreting Figure 1.

L307-309: Please explain what the "simple spatial constraints" are, as it is difficult to interpret this as written.

L312: This appears to be a typo: "?" where a figure reference should be?

© 2026. Published by The Company of Biologists under the terms of the Creative Commons Attribution License (https://creativecommons.org/licenses/by/4.0/).

L314-316: How were these performance metrics evaluated? Presumably against a ground truth or manual annotation? This does not appear to be described in the Materials and Methods section.

L364-381: This appears to be primarily a comparison of this automated system to conventional wooden hives, but I believe there are other examples of fabricated hives, for example plastic, that would seem to be the most relevant comparison.

L380-381: Are these prices for the entire setup here, including tracking frame, etc., or just for the frame materials?

L382-388: These results are not described in the Results section. How were brood establishment, survival, etc. assessed?

L390: It is not clear to me what "high-precision" means here.

L393: I think "modularity and adaptability" need to be described more specifically. What makes this system easier to modify than other designs, wooden or otherwise? That has not yet been clearly demonstrated, but it is highlighted as a central claim of the advance here.

L463: It would be helpful to add text labels to the dotted figure outlines in Figure 2B.

L501: Another "?" here that appears to be a typo.

L503-504: Is this a constraint of the system, or just the focus of the validation? That is, could the system be used to track multiple bees?

L562-564: How was this ratio derived? By comparing the two methods on a common dataset?

Reviewer's Responses to Questions

Experimental quality
Does each figure have the proper controls?
If 'No', please indicate reasons in Comments for Author box below.
Reviewer #1:
- Yes

Reviewer #2:
- Yes

Were the data analyzed using appropriate statistical tests?
If 'No', please indicate reasons in Comments for Author box below.
Reviewer #1:
- Yes

Reviewer #2:
- No

Reproducibility
Were experiments performed using adequate number of biological replicates?
If 'No', please indicate reasons in Comments for Author box below.
Reviewer #1:
- Yes

Reviewer #2:
- Yes

© 2026. Published by The Company of Biologists under the terms of the Creative Commons Attribution License (https://creativecommons.org/licenses/by/4.0/).

Does the methods section provide sufficient detail to permit reproducibility?
If 'No', please indicate reasons in Comments for Author box below.
Reviewer #1:
- Yes

Reviewer #2:
- Yes

Completeness
Are the manuscript's conclusions supported by the data?
If 'No', please indicate reasons in Comments for Author box below.
Reviewer #1:
- Yes

Reviewer #2:
- No

Scholarship
Do the authors cite and discuss the merits of data that would argue for and against their conclusion?
If 'No', please indicate reasons in Comments for Author box below.
Reviewer #1:
- Yes

Reviewer #2:
- Yes

Does the manuscript title & abstract accurately reflect the contents of the manuscript, without hyperbole?
If 'No', please indicate reasons in Comments for Author box below.
Reviewer #1:
- Yes

Reviewer #2:
- Yes

First revision

Author response to reviewers' comments

Dear Dr Greenlee,

We thank you and both reviewers for the constructive and detailed feedback on our manuscript. We are encouraged by the positive overall assessment and have worked carefully through every comment. The major revisions include:

1. Substantial expansion of the Introduction to provide concrete technical descriptions of prior automated tracking systems (camera resolutions, distances, lighting configurations, marker vs. markerless approaches) and to articulate the specific standardisation, modularity, and biocompatibility limitations our system addresses.
2. A new paragraph previewing the validation framework, biological (colony health monitoring across six colonies over two months) and technical (continuous queen tracking with WhyComb).

© 2026. Published by The Company of Biologists under the terms of the Creative Commons Attribution License (https://creativecommons.org/licenses/by/4.0/).

3. Improved statistical reporting throughout the Results section, including test statistics alongside all p-values, correct labelling of test statistics (U, H, τ), and clarification of which comparisons are descriptive versus formally tested.
4. A more concrete explanation of the system's modularity.
5. Expanded discussion of the relationship between adult population size and colony health, including the biological reasoning for treating population stability as a summer-specific health proxy and the limitations of this proxy with respect to detectability drift.
6. A new Results paragraph reporting the qualitative weekly inspections by professional apiarists, complementing the quantitative population data.

Detailed point-by-point responses follow. Reviewer comments are quoted in full, our responses are provided in plain text.

Reviewer 1 summary: This manuscript aims at reporting an open-access technology for tracking bees in an observation hive. This is certainly of interest for the field and the methods are easy-to-follow and sufficiently described to be re-used by other labs. I particularly appreciated the effort made to evaluate the budget. I therefore have only a few recommendations to improve the readiness of the need for a standardized methodology as claimed by the authors but also its potential applications.

R1-1: Thank you for the table 1 listing the main bibliography of the topic. However, it requires quite an effort to make sense of it. Would it be possible to add information about e.g. the main focus/question of the study, the level of technology involved and in particular for the last part, most relevant for the current paper, highlighting the differences between the different setup ?

Answer: We agree that listing the bibliography alone does not effectively communicate the methodological differences across the literature. We have significantly expanded the introduction to explicitly discuss key publications highlighted in the table's "Automated" section. The new text details the specific technologies utilized (e.g., camera resolutions, marker vs. markerless tracking) and contrasts their physical setups (e.g., varying camera distances and lighting angles).

R1-2: Could you better illustrate the diversity of methods used in different studies and why this may have an impact on results ?

Answer: We have updated the Introduction to explicitly describe diverse sensor rigging and hardware constraints used in recent automated studies. The new text clarifies how these disparate optical and physical setups limit the transferability of computer vision models.

R1-3: I understand only at the end of the discussion the purpose of table 2 (counting bees) in particular as you seemed not have used your automatic tracking system to this end. If I am correct, this aimed at evaluating mortality in your observation hive ? This should be better explained in the table caption and the results section.

Answer: We agree with this assessment, the bee counts were conducted to evaluate mortality and establish the biocompatibility of the hive, and this was not communicated early enough in the text. We have updated the captions for Tables 2 and 3 to explicitly state this purpose. Furthermore, we have expanded the opening paragraph of the "Colony Health Assessment" in the Results section to clearly define this biological objective before presenting the statistics.

R1-4: As it stands, your software can only track the queen as far as I understood. Could you comment on potential add-ons, maybe with other tracking systems from different studies that may exist as open-source ? For example, would it be possible to follow individual workers with your labeling systems ? How many in parallel ? Would it be possible to overcome the issue of bees overlapping at it impacted the automatic counting apparently ? Finally, would the software need to be trained with labeled frames to be able to track the queen, if yes could you quantity and explicit the necessary work ?

Answer: We appreciate the reviewer's thoughtful questions regarding the system's tracking

© 2026. Published by The Company of Biologists under the terms of the Creative Commons Attribution License (https://creativecommons.org/licenses/by/4.0/).

capabilities and potential extensions. We have expanded the Tracking System section to address each of these points in detail.
Regarding worker bee tracking: While our current validation focuses on queen tracking, the system can indeed be adapted to track individual worker bees using the same computer vision pipeline. However, this would require marking each worker with fiducial markers similar to those used for the queen, which presents practical challenges. Worker bees are physically smaller than queens, necessitating smaller markers and correspondingly higher camera resolution to maintain detection accuracy. Additionally, workers frequently leave the hive for foraging flights, increasing the risk of marker loss. The number of bees that could be tracked in parallel would depend on the camera resolution and computational resources available.
Regarding overlapping bees and automatic counting: The reviewer correctly identifies bee overlapping as a challenge for population estimation. We have added discussion of potential solutions, including reducing the distance between glass panels and wax combs to limit vertical bee movement, or using artificial combs with more even surfaces to prevent local valleys where bees tend to cluster. The automatic counting method (Ulrich et al., 2024) uses a convolutional neural network that does not count partially visible bees, which we addressed by deriving a scaling ratio (1.418) from manual annotation of three representative days.
Regarding training requirements: The queen-tracking method employed (WhyComb, Žampachů et al., 2022) is based on fiducial marker detection using known geometric properties of perspective projection, which does not require training with labeled frames. However, worker bee detection and population counting rely on a convolutional neural network trained in a supervised manner, which would require several thousand annotated images for adequate performance.

Reviewer 2 summary: This is an interesting and potentially valuable methods paper. The overall concept is strong, and the manuscript is framed around an important problem for the field: the need for more standardised, reproducible platforms for automated honeybee observation. The emphasis on open hardware, compatibility with existing beekeeping practice, and the goal of improving comparability across studies are all compelling. I also think the broader motivation is timely, given the increasing use of computer vision and automated tracking in behavioural and applied honeybee research. However, I think there are a few key issues with the manuscript that need to be addressed.

R2-1: First, the novelty of this system relative to existing systems, especially for automated tracking, is not well described. For example, the introduction discusses identifying and addressing shortcomings of existing systems, including developing a system that is "modular and adaptable." However, the description of these other systems is vague, so it is hard to assess what the specific existing shortcomings are or how the system presented here addresses them. In addition, it is not entirely clear how the challenges of modularity and adaptability are addressed in practice. What is the range of research applications for which this system could potentially be used, and what makes it more adaptable to a wider range of applications than other automated systems? Is this primarily because it is open source, and thus modifiable? Are there other practical barriers that limit reproducibility and standardisation? Relatedly, some of the key terms used here, such as "precision" and "reproducibility," are a bit vague, and the manuscript would benefit from clarifying what the authors mean by these terms in this context.

Answer: We have significantly revised the Introduction to provide concrete, detailed descriptions of the existing automated tracking setups used in recent literature. This new text explicitly outlines the practical barriers, biological compromises, and lack of standardisation in current bespoke methodologies that our open-hardware system is designed to address.

R2-2: Second, the example validation dataset is not sufficiently described. There are several issues with the reporting of statistics and results, as noted below. More importantly, many key details, including the analyses, validation procedures, performance metrics, and biological outcome measures, are not described clearly enough in the current Results section or previewed in the Introduction. This makes it difficult to assess what was measured, how it was validated, and how strongly the presented data support the manuscript's conclusions. While some of these details can be found in the Materials and Methods, at least high-level descriptions should be included earlier in the manuscript to provide readers with context for understanding the capabilities of the system.

© 2026. Published by The Company of Biologists under the terms of the Creative Commons Attribution License (https://creativecommons.org/licenses/by/4.0/).

Answer: We sincerely thank the reviewer for this valuable feedback regarding the presentation of our validation framework. We agree that a clearer upfront description of the validation approach strengthens the manuscript and helps readers properly contextualise the results. We have implemented the following revisions:
First, in the Introduction, we added a new paragraph that previews the validation framework: (1) biological validation through colony health monitoring over approximately two months during the active foraging season, assessing population stability across six colonies; and (2) technical validation through demonstration of continuous queen tracking capability using the WhyComb system.
Second, we expanded the introductory text of the Results section to explicitly outline the validation scope before presenting specific statistical outcomes.
Third, we have improved the clarity of statistical reporting throughout the Results section by adding test statistics alongside p-values, specifying the statistical tests used, and clarifying which comparisons were descriptive versus formally tested.
Fourth: We extended the discussion by an interpretation of the validation procedure.

R2-3: Finally, the potential applications of this system are not well described. In particular, what types of research, whether basic or applied, would be enabled by this system that are not yet possible? Providing a few concrete examples would help readers understand the benefits of the system and its potential for broader adoption.

Answer: The manuscript has been revised to provide concrete examples of how the system's engineering advantages translate into biological research applications. We have amended the Introduction to clarify that while automation should enable higher throughput replication, the difficulty of precisely standardizing and calibrating the entire observation setup (including exact camera distances and lighting angles) using traditional wooden frames limits many studies to single-colony proofs-of-concept. This lack of optical uniformity in ad-hoc setups forces resource-intensive model retraining across different datasets.

Furthermore, we have added a dedicated paragraph to the Discussion section outlining three specific applications uniquely enabled by the precise tolerances of our aluminium-profile system:

1. Scalable, multi-colony replication for assessing environmental stressors, as the aluminium profiles allow all optical distances to be replicated exactly across setups;
2. Standardized multi-site studies where computer vision models can be shared across laboratories without retraining due to guaranteed optical uniformity; and
3. Multi-generational pathogen and toxicological studies permitted by the sterilizable frame.

R2-4: L15-197: Important to note here that honeybee populations are not declining per se; there are high annual losses, but total colony numbers are generally stable or potentially increasing.

Answer: Thank you for this correction. We have amended the Abstract to accurately refer to "high annual colony losses" rather than a population decline.
R2-5: L98: The use of "rigour" here seems a bit vague. These tools could certainly be used for experiments we would not call rigorous, and traditional approaches can also be highly rigorous. Rephrase?

Answer: The relevant section has been completely revised to clarify the point and prevent any confusion.

R2-6: L154: Does "comparative" here mean multiple species, or comparison across conditions, experimental setups, etc.? Please expand.

Answer: Thank you for pointing out this ambiguity. While the original sentence in the Introduction was removed during our structural revisions, we have explicitly clarified this concept in a newly added paragraph in the Discussion section. We now specify that "comparative studies" in this context refers to comparing honeybee behaviours across different geographical

© 2026. Published by The Company of Biologists under the terms of the Creative Commons Attribution License (https://creativecommons.org/licenses/by/4.0/).

locations, climates, or local environmental conditions across multiple laboratory sites. By standardizing the physical and optical parameters across these disparate sites, laboratories can directly share and apply pre-trained computer vision models. This can eliminate the need for resource-intensive local retraining, removing a major technical bottleneck in collaborative ethological research.

R2-7: L156-167+: Table 1 does not specify what these limitations are, so it would be helpful to articulate them, as this is central to describing the novelty of the system relative to other automated honeybee tracking systems. In particular, the authors emphasise the system as being "modular and adaptable," but it is not clear to what extent this is true, or not true, for existing systems, or how the approach here differs.

Answer: We have rewritten the corresponding narrative in the Introduction and tried to clarify the point in the discussion as well. The new text explicitly articulates the specific hardware limitations, operational disruptions, and standardisation issues present in existing systems, as well as the modularity of the new system.

R2-8: L165: "uses the widely used ..." is repetitive.

Answer: We thank the reviewer for identifying this redundancy. We have revised the text to eliminate the repetitive phrasing "uses the widely used". The sentence now reads "follows a historically established research format and uses the Zander comb size, ensuring immediate compatibility with standard beekeeping practices in Austria"

R2-9: L209-211: A bit vague. Does the current setup work for, for example, Langstroth hives? If not, what would need to be changed?

Answer: We agree that the practical mechanism of the system's modularity was vaguely described. We have expanded this section to explicitly use the Langstroth frame as an example of how the system adapts to different formats. The text now details that switching from a Zander frame to a Langstroth frame only requires adjusting the cut lengths of the standard horizontal and vertical aluminium profiles, without necessitating any redesign of the structural joints or sensor mounts.

R2-10: L255: The connection between this estimate of the number of bees in a hive, presumably from tracked data, though that is not actually very clear from the text, and colony health is not very clear. Is it also possible to disentangle changes driven by tracking performance, for example tag loss or detectability effects, from true population effects here?

Answer: We have clarified in the text that the population size estimates were derived from images: manual bee annotation (2023 data) and automatic bee detection using a convolutional neural network (2024 data). The fiducial marker was used only for queen tracking and does not affect population estimates. To address the connection to colony health, we assessed colony health primarily through population stability over time. Healthy colonies maintain relatively stable populations (low coefficients of variation) without significant declining trends. We quantified this using temporal trend analysis (Theil-Sen slope, Kendall's τ) and inter-week fluctuation tests. Colonies with low CV values and no significant negative trends were considered healthy, while substantial population decline would indicate potential health issues. Additionally, we monitored brood production and general vigour as supplementary health indicators. Regarding neural network detection performance, an underestimation was corrected by manually annotating three days (June 30, July 29, and August 19) of colony C to derive a scaling ratio (1.418), ensuring accurate population estimates. We have also extended the discussion to highlight the connection between the estimate of the number of bees in a hive and colony health.

R2-11: L259-260: Is there a statistical test of this difference? Similar comments apply to other comparisons below.

Answer: We appreciate the reviewer's careful attention to our statistical reporting. The reviewer is correct to question whether formal statistical testing was conducted for the comparisons of the coefficient of variation. We have revised the text to remove language that implied statistically

© 2026. Published by The Company of Biologists under the terms of the Creative Commons Attribution License (https://creativecommons.org/licenses/by/4.0/).

tested differences between colonies A and B, as these CV values were intended solely as descriptive observations of population stability rather than formal comparative statistics. The coefficients of variation serve to characterise the temporal variability within each colony independently, and we have clarified this descriptive purpose in the Results section. Where formal comparisons were made (e.g., inter-colony population distributions), we have ensured that appropriate statistical tests and test statistics are now reported throughout the manuscript.

R2-12: L260: Similarly, is there a statistical test for what is presumably a linear model?

Answer: We thank the reviewer for this opportunity to clarify our methodological approach. We have revised the text to explicitly state that we used the Theil-Sen slope estimator rather than a traditional linear regression model. The Theil-Sen estimator is a non-parametric method that calculates the median slope among all pairs of sample points, making it more robust to outliers and appropriate for our small sample sizes, where parametric assumptions may not hold. We have added this clarification to the Results section.

R2-13: L298-299: Test statistics should be reported in addition to p-values.
Answer: We appreciate the reviewer's recommendation for more complete statistical reporting. We have now added the test statistics alongside all p-values throughout the Results section.

R2-14: L302-305: It is not clear what "qualitative tracking" means here. The WhyComb system needs to be described in more detail here, or at least its outputs and capabilities as relevant for interpreting Figure 1.

Answer: We thank the reviewer for this valuable suggestion to clarify our tracking methodology. We have revised the text to explain that "qualitative tracking" refers to demonstrating the general capability and feasibility of continuous queen tracking within the hive, rather than providing a comprehensive quantitative analysis of movement dynamics. The WhyComb system outputs the queen's position in time within the hive-defined coordinate system, together with a small cropped image of the queen's immediate vicinity to reduce bandwidth and storage requirements as described in (Žampachů et al., 2022).

R2-15: L307-309: Please explain what the "simple spatial constraints" are, as it is difficult to interpret this as written.

Answer: We appreciate the reviewer's request for clarification regarding our trajectory filtering approach. We have expanded the text to explicitly describe the spatial constraints applied to avoid false detections. Specifically, we allowed detections only within the physically accessible area of the hive, defined by the coordinate bounds, which correspond to the actual comb surface area accessible to the queen. Additionally, we applied detector parameter constraints to ensure detection confidence thresholds were met. We also split continuous tracking into smaller sequences whenever a detection jumped by more than 3 cm, and retained only sequences containing at least 20 detections to ensure trajectory continuity. These constraints are now fully described in the Queen Tracking section to improve methodological transparency.

R2-16: L312: This appears to be a typo: "?" where a figure reference should be?

Answer: We thank the reviewer for identifying this typographical error. The "?" symbol was indeed a placeholder that was inadvertently left in the manuscript during the revision process. We have corrected this by replacing the "?" with the proper reference to Blaha et al. (2025), which contains the detailed movement dynamics analysis.

R2-17: L314-316: How were these performance metrics evaluated? Presumably against a ground truth or manual annotation? This does not appear to be described in the Materials and Methods section.

Answer: We appreciate the reviewer's question regarding the evaluation of our tracking performance metrics. The localisation error (4.3 pixels), detection precision (0.987), and recall (1.0) values reported in the manuscript originate from the referenced publication Žampachů et

© 2026. Published by The Company of Biologists under the terms of the Creative Commons Attribution License (https://creativecommons.org/licenses/by/4.0/).

al. (2022), which provides a comprehensive description of the WhyComb tracking system's development and validation. In that publication, these metrics were evaluated against manually annotated ground truth data from honeybee hive images, with precision and recall calculated based on the system's ability to correctly identify and localize a fiducial marker attached to a honeybee queen. We have revised the text to explicitly cite this source and clarify that the performance validation methodology is described in detail in Žampachů et al. (2022), rather than duplicating this information in the current manuscript. This approach allows us to focus on the novel hive design while directing readers to the appropriate reference for tracking system performance details.

R2-18: L364-381: This appears to be primarily a comparison of this automated system to conventional wooden hives, but I believe there are other examples of fabricated hives, for example plastic, that would seem to be the most relevant comparison.

Answer: We appreciate this point. While wooden observation hives have historically been the standard for research, fabricated plastic and acrylic display hives do exist. We have amended the text to include these fabricated materials in our comparison. Specifically, we expanded the "Modularity" and "Durability" bullet points to highlight that while plastic hives resolve some issues inherent to wood, they still lack the non-destructive adaptability of T-slot aluminium (requiring drilling for modifications) and are susceptible to degradation from UV exposure or cleaning solvents over time.

R2-19: L380-381: Are these prices for the entire setup here, including tracking frame, etc., or just for the frame materials?

Answer: We thank the reviewer for raising this important clarification regarding the cost breakdown. We have revised the text to explicitly state that the quoted prices (135 € for aluminium, 260 € for wooden hives) refer to the hive frame construction only, and do not include additional components such as cameras, sensors, lighting systems, or their mounting frames. We acknowledge that any complete experimental setup would require these extensions regardless of whether the hive is constructed from aluminium or wood, and we assume the cost of these components remains relatively consistent across both hive types. We have added a clarifying note in the Discussion section to ensure readers understand that the total system cost will be higher when accounting for the full observation and tracking infrastructure, which varies depending on the specific experimental requirements and equipment choices.

R2-20: L382-388: These results are not described in the Results section. How were brood establishment, survival, etc. assessed?

Answer: This is a very valid point, our original phrasing incorrectly implied that formal quantitative assays of brood development and individual survival rates were conducted. We have revised the manuscript to accurately reflect our methodology for assessing biocompatibility. First, we expanded the Methods section to formally detail the weekly qualitative health inspections conducted by professional apiarists to verify normal brood rearing and absence of disease. Second, we revised the Discussion section to clarify that continuous "brood establishment" and "survival" are implicitly confirmed by the stable population sizes recorded over the two-month period (detailed in Tables 2 and 3), as stable summer populations are biologically impossible without continuous brood replacement.

R2-21: L390: It is not clear to me what "high-precision" means here.
Answer: We agree that the term "high-precision" was used ambiguously in the concluding paragraph. We have revised the text to explicitly define this term in an engineering context. The manuscript now states that the framework leverages the "high-precision manufacturing tolerances of standard aluminium extrusions" for better structural and optical reproducibility of the setups.

R2-22: L393: I think "modularity and adaptability" need to be described more specifically. What makes this system easier to modify than other designs, wooden or otherwise? That has not yet been clearly demonstrated, but it is highlighted as a central claim of the advance here.

© 2026. Published by The Company of Biologists under the terms of the Creative Commons Attribution License (https://creativecommons.org/licenses/by/4.0/).

Answer: We have revised the "Modularity" bullet point in the Discussion to provide concrete engineering specifics. The text now explains that the system’s use of T-slot aluminium provides non-destructive adaptability. This allows equipment to be attached, repositioned, and removed.

R2-23: L463: It would be helpful to add text labels to the dotted figure outlines in Figure 2B.

Answer: We thank the reviewer for this helpful suggestion. We have now added clear text labels to both Figures 2A and 2B to identify the key components of the observation system. These labels correspond to the sensor frame (I), lighting frame (II), and feeder (III), which are now explicitly referenced in the figure caption and the main text. We believe these additions significantly improve the figure's clarity and make it easier for readers to understand the system architecture at a glance.

R2-24: L501: Another "?" here that appears to be a typo.

Answer: We appreciate the reviewer's careful attention to detail. This placeholder symbol was unintentional and resulted from an incomplete citation during manuscript preparation. We have now corrected this by replacing the "?" with the proper reference (Zhang, 2002) for the camera calibration chessboard pattern method. The reference list has been updated accordingly to ensure complete and accurate citation throughout the manuscript.

R2-25: L503-504: Is this a constraint of the system, or just the focus of the validation? That is, could the system be used to track multiple bees?

Answer: We thank the reviewer for raising this important point, which allows us to clarify the system's capabilities. The focus on queen tracking in this study was a deliberate choice for validation purposes, as the queen represents an ideal focal subject for demonstrating continuous, long-term observation within the hive. This is not a fundamental constraint of the system itself. The same computer vision pipeline and hardware setup can be adapted to track multiple worker bees, provided that individual bees are marked with fiducial markers and the camera resolution is increased to accommodate the smaller marker size required for workers. We have expanded the Tracking System section to explicitly discuss this scalability and the practical considerations involved in multi-bee tracking, including the additional labour required for marking and the potential for marker or bee loss during foraging flights.
R2-26: L562-564: How was this ratio derived? By comparing the two methods on a common dataset?

Answer: We appreciate the reviewer's request for clarification on this methodological detail. Yes, the scaling ratio (1.418) was derived by directly comparing manual human annotation with automated bee counting on the same image dataset. Specifically, a trained human annotator manually counted bees in images from three representative days (June 30, July 29, and August 19, 2024) from colony C. These counts were then compared to the population estimates obtained from the same images using the automated convolutional neural network method (Ulrich et al., 2024). The ratio represents the mean proportion of human-annotated counts to automated counts across these three days. This calibration step was necessary because the automated method does not count partially visible bees, leading to systematic underestimation. We have added this explanation to the Biological Validation section to improve methodological transparency.

<u>Second decision letter</u>

MS ID#: bio.062523R1

MS Title: An Open-Source High-precision Hive for Long-Term Honeybee Observation and Research

© 2026. Published by The Company of Biologists under the terms of the Creative Commons Attribution License (https://creativecommons.org/licenses/by/4.0/).

Authors: Jiří Ulrich, Martin Stefanec, Tomáš Rouček, Laurenz Alexander Fedotoff, Harald Pascher, Zdeněk Rozsypálek, Thomas Schmickl and Tomáš Krajník

I have now reached a decision on the above manuscript.

The reviewer reports are shown at the bottom of this email.

As you will see, one of the reviewers had a few additional comments. I agree with the reviewer, especially regarding the additional labeling on the figures and information about the feasibility of modifying the system to track multiple workers. I hope that you can make these changes, because I would like to accept your paper.

At this stage, we also ask you to ensure your manuscript complies with our formatting guidelines - please see our manuscript preparation guidelines for details. Provided you are able to fully address the referees' comments, we are positive about publication of your paper (we accept over 95% of revision submissions) and therefore hope you won't mind any extra work involved in reformatting your manuscript at this point.

Please upload both a 'clean' version of your Word file, along with a highlighted version clearly showing where you have made changes in the revised manuscript. Please avoid using 'Track changes' in Word files as these are lost in PDF conversion.

I should be grateful if you would also provide a point-by-point response detailing how you have dealt with the points raised by the reviewers in the 'Response to Reviewers' box. Please attend to all of the reviewers' comments. If you do not agree with any of their criticisms or suggestions please explain clearly why this is so.

Reviewer 1

Comments for the author

The authors have adequately addressed my comments and suggestions. The manuscript is now suitable for publication.

Reviewer 2

Comments for the author

Overall, the edited manuscript is much improved, and in particular the additional text in the introduction and discussion will I think be very helpful for readers in understanding the novelty and potential utility of the system developed here, as well as how it advances on existing approaches. I commend the authors on their careful revisions and significant improvements to the manuscript, and I have only some minor comments remaining

L105: Minor, but I would suggest rewording 'strict requirement' to something like 'it is increasingly important'

Figure 1: Potentially just an issue with my version, but the resolution on Figure 1 is quite low. And up to the authors, but I would suggest adding text labels onto the figures themselves, in addition to just numerals, to aid interpretation

L188: Maybe define 'CAD' here or just describe as 3D model rendering.

L227: Perhaps switch different 'logic' for different 'pattern'?

L389-392: I suspect tracking multiple workers would potentially be a primary use case of interest to researchers - it might be worth expanding this section to explore how feasible this kind of modification would be (if feasible at all) from the current system.

Reviewer's Responses to Questions

© 2026. Published by The Company of Biologists under the terms of the Creative Commons Attribution License (https://creativecommons.org/licenses/by/4.0/).

Experimental quality
Are experiments performed using appropriate methods that will answer the question (or test the hypothesis or support the observations) posed by the authors? Is the right tool used for the job?
If 'No', please indicate reasons in Comments for Author box below.
Reviewer #1:
- Yes

Reviewer #2:
- Yes

Were the data analyzed using appropriate statistical tests?
If 'No', please indicate reasons in Comments for Author box below.
Reviewer #1:
- Yes

Reviewer #2:
- Yes

Reproducibility
Were experiments performed using adequate number of biological replicates?
If 'No', please indicate reasons in Comments for Author box below.
Reviewer #1:
- Yes

Reviewer #2:
- Yes

Is there sufficient raw data to assess the rigor of the analysis?
If 'No', please indicate reasons in Comments for Author box below.
Reviewer #1:
- Yes

Reviewer #2:
- Yes

Does the methods section provide sufficient detail to permit reproducibility?
If 'No', please indicate reasons in Comments for Author box below.
Reviewer #1:
- Yes

Reviewer #2:
- Yes

Completeness
Are the manuscript's conclusions supported by the data?
If 'No', please indicate reasons in Comments for Author box below.
Reviewer #1:
- Yes

Reviewer #2:
- Yes

© 2026. Published by The Company of Biologists under the terms of the Creative Commons Attribution License (https://creativecommons.org/licenses/by/4.0/).

Scholarship
Do the authors cite and discuss the merits of data that would argue for and against their conclusion?
If 'No', please indicate reasons in Comments for Author box below.
Reviewer #1:
- Yes

Reviewer #2:
- Yes

Does the manuscript title & abstract accurately reflect the contents of the manuscript, without hyperbole?
If 'No', please indicate reasons in Comments for Author box below.
Reviewer #1:
- Yes

Reviewer #2:
- Yes

Second revision

Author response to reviewers' comments

Dear Dr Greenlee,

Thank you for the positive decision on our manuscript and for the opportunity to make final revisions. We are grateful for the constructive feedback from Reviewer 2 and the additional guidance in your letter. We have carefully addressed every remaining point.

We would also like to bring one further matter to your attention. Currently, data are shared via the authors' cloud hosting. We plan to move them to a persistent, citable Zenodo repository before publication. The data availability statement and the corresponding link in the manuscript will therefore need to be updated accordingly. We can provide the Zenodo link at the proof stage.

We believe the manuscript is now fully ready for publication. Point-by-point responses to all reviewer comments are included below.

Reviewer 1 summary: The authors have adequately addressed my comments and suggestions. The manuscript is now suitable for publication.

Answer: We thank the reviewer for the time and effort spent evaluating our manuscript. We are pleased that the revisions have addressed the earlier comments and that the reviewer now recommends publication of the manuscript.

Reviewer 2 summary: Overall, the edited manuscript is much improved, and in particular the additional text in the introduction and discussion will I think be very helpful for readers in understanding the novelty and potential utility of the system developed here, as well as how it advances on existing approaches. I commend the authors on their careful revisions and significant improvements to the manuscript, and I have only some minor comments remaining

R2-1: Figure 1: Potentially just an issue with my version, but the resolution on Figure 1 is quite low. And up to the authors, but I would suggest adding text labels onto the figures themselves, in addition to just numerals, to aid interpretation

Answer: The figure has been regenerated at a higher resolution, and textual labels have now been

© 2026. Published by The Company of Biologists under the terms of the Creative Commons Attribution License (https://creativecommons.org/licenses/by/4.0/).

added directly to the figure alongside the numerals. This makes the setup easier to interpret at a glance.

R2-2: L105: Minor, but I would suggest rewording 'strict requirement' to something like 'it is increasingly important'
L188: Maybe define 'CAD' here or just describe as 3D model rendering. L227:
Perhaps switch different 'logic' for different 'pattern'?

Answer: We thank the reviewer for the thoughtful comments. All suggested wording changes have been incorporated into the revised manuscript to improve clarity and delivery.

R2-3: L389-392: I suspect tracking multiple workers would potentially be a primary use case of interest to researchers - it might be worth expanding this section to explore how feasible this kind of modification would be (if feasible at all) from the current system.

Answer: We have elaborated on the multiple-worker tracking scenarios, outlining which systems to use for which number of tracked individuals.

Third decision letter

MS ID#: bio.062523R2

MS Title: An Open-Source High-precision Hive for Long-Term Honeybee Observation and Research

Authors: Jiří Ulrich, Martin Stefanec, Tomáš Rouček, Laurenz Alexander Fedotoff, Harald Pascher, Zdeněk Rozsypálek, Thomas Schmickl and Tomáš Krajník

Thank you for making the recommended changes to your manuscript. I am happy to tell you that your manuscript has been accepted for publication in Biology Open, pending our standard publication integrity checks. It was accepted on 19th May 2026. You noted in your response that the link to the data repository would be updated, and it is fine to do that in the proof stage.

© 2026. Published by The Company of Biologists under the terms of the Creative Commons Attribution License (https://creativecommons.org/licenses/by/4.0/).
